# Clinical Guideline for Preimplantation Genetic Testing in Inherited Cardiac Diseases

**DOI:** 10.1161/CIRCGEN.123.004416

**Published:** 2024-03-22

**Authors:** Job A.J. Verdonschot, Debby M.E.I. Hellebrekers, Vanessa P.M. van Empel, Malou Heijligers, Sonja de Munnik, Edith Coonen, Jos C.M.F. Dreesen, Arthur van den Wijngaard, Han G. Brunner, Masoud Zamani Esteki, Stephane R.B. Heymans, Christine E.M. de Die-Smulders, Aimée D.C. Paulussen

**Affiliations:** Department of Clinical Genetics, Maastricht University Medical Center, the Netherlands (J.A.J.V., D.M.E.I.H., M.H., S.d.M., E.C., J.C.M.F.D., A.v.d.W., H.G.B., M.Z.E., C.E.M.d.D.-S., A.D.C.P.).; Department of Cardiology, Maastricht University, Cardiovascular Research Institute Maastricht, the Netherlands (J.A.J.V., V.P.M.v.E., S.R.B.H.).; European Reference Network for Rare, Low Prevalence and Complex Diseases of the Heart (ERN GUARD-Heart) (J.A.J.V., D.M.E.I.H., V.P.M.v.E., S.R.B.H.).; Department of Human Genetics, Donders Institute for Brain, Cognition and Behavior, Radboud University Medical Center, Nijmegen, the Netherlands (S.d.M., H.G.B.).; GROW School for Oncology and Reproduction, Maastricht University, the Netherlands (E.C., H.G.B., M.Z.E., C.E.M.d.D.-S., A.D.C.P.).; Department of Cardiovascular Sciences, Centre for Molecular and Vascular Biology, KU Leuven, Belgium (S.R.B.H.).

**Keywords:** American Heart Association, Brugada syndrome, cardiomyopathies, heart disease, heart transplantation

## Abstract

**BACKGROUND:**

Preimplantation genetic testing (PGT) is a reproductive technology that selects embryos without (familial) genetic variants. PGT has been applied in inherited cardiac disease and is included in the latest American Heart Association/American College of Cardiology guidelines. However, guidelines selecting eligible couples who will have the strongest risk reduction most from PGT are lacking. We developed an objective decision model to select eligibility for PGT and compared its results with those from a multidisciplinary team.

**METHODS:**

All couples with an inherited cardiac disease referred to the national PGT center were included. A multidisciplinary team approved or rejected the indication based on clinical and genetic information. We developed a decision model based on published risk prediction models and literature, to evaluate the severity of the cardiac phenotype and the penetrance of the familial variant in referred patients. The outcomes of the model and the multidisciplinary team were compared in a blinded fashion.

**RESULTS:**

Eighty-three couples were referred for PGT (1997–2022), comprising 19 different genes for 8 different inherited cardiac diseases (cardiomyopathies and arrhythmias). Using our model and proposed cutoff values, a definitive decision was reached for 76 (92%) couples, aligning with 95% of the multidisciplinary team decisions. In a prospective cohort of 11 couples, we showed the clinical applicability of the model to select couples most eligible for PGT.

**CONCLUSIONS:**

The number of PGT requests for inherited cardiac diseases increases rapidly, without the availability of specific guidelines. We propose a 2-step decision model that helps select couples with the highest risk reduction for cardiac disease in their offspring after PGT.

Preimplantation genetic testing (PGT) is an assisted reproductive technology that is used to select embryos without (familial) genetic variants.^1^ PGT can be offered to couples at high risk of conceiving a child with a monogenic disorder or a structural chromosome rearrangement.^2^ Selecting unaffected embryos requires an in vitro fertilization treatment with intracytoplasmic sperm injection followed by genetic testing of biopsied embryonic cells. The impact of a PGT trajectory should not be underestimated, as the physical burden for the woman, and the psychological impact for the couple are high. Also, the procedure is associated with significant costs. The procedure may take many months and is not always successful in leading to a pregnancy and the birth of a healthy baby (≈50%–60% of women, PGT is successful). Couples need to be counseled about the expected costs of the procedure, the success rates, the duration of the trajectory, and the procedure of in vitro fertilization.

The initial rationale for PGT has been to prevent young onset, severe diseases with complete penetrance.^1^ In contrast, inherited cardiomyopathies and arrhythmias are mostly adult-onset, with variable expression and incomplete penetrance. Most inherited cardiac diseases are autosomal dominant, thus, the child has 50% of inheriting the familial variant. Characterization of the pathogenic variant, and the phenotype in a family, is important to determine the causality of the variant in the family and the severity of the familiar phenotype and make a valid selection of couples at high risk for conceiving a child with overt cardiac disease. Unnecessary PGT treatments for genetic variants with variable disease expression and low penetrance should be prevented to avoid long trajectories, burden, and costs for young couples, while the risk reduction for their offspring is probably low or unclear. The possibilities, strategies, ethics, moral opinions, and laws around PGT strongly differ among countries, and the disease severity of inherited cardiac diseases may be differently perceived by couples based on personal and family experience.^3^ The absence of a global policy or common guideline and national or local preferences, may lead to an inequality in accessibility and affordability of PGT for couples with inherited cardiac diseases. However, the demand for PGT in general is increasing, and the availability of PGT for inherited cardiac diseases is broadening. An objective decision model based on clinical and genetic variables can help to guide clinicians to discuss the expected risk reduction by PGT with their patients, thereby increasing accessibility. The latest guidelines of American (American College of Cardiology and American Heart Association [AHA]) and European (European Society of Cardiology and European Heart Rhythm Association) cardiology societies listed PGT as an option for patients with monogenic heart diseases in childbearing age, acknowledging the absence of strategy guidelines of PGT for cardiogenetic indications.^4–7^ In the current study, we retrospectively reviewed the clinical information of couples with an inherited cardiac disease who were referred to the national PGT center in the past 25 years. Furthermore, we aimed to validate a new decision model based on the most recent published cardiac risk prediction models and genetic curation literature, which can be used to assess the penetrance of variants in families, and subsequent admissibility of PGT referrals for inherited cardiac diseases (Figure 1; Supplemental Methods). This model can provide guidance to the treating physician in the counseling of couples with an inherited cardiac disease and a child wish.

**Figure 1.**
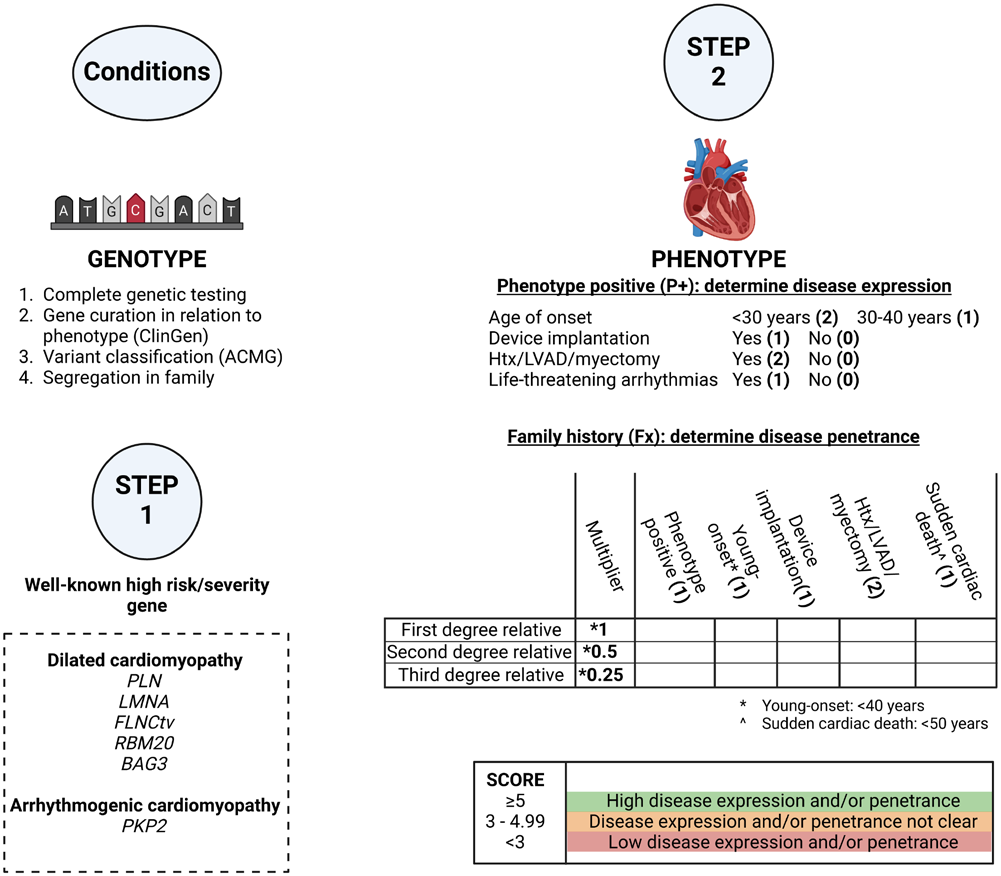
**Two-step decision model to evaluate preimplantation genetic testing (PGT) referrals for inherited cardiac diseases.** See Methods section for detailed description of the conditions and 2 steps. The numbers in bold behind the clinical parameters in step 2 indicate the number of points that can be scored (see Figure 4 for 2 examples of application of the model). A first degree relative (50% shared DNA) is an individual’s parent, full sibling, or child. Second degree (25% shared DNA) relatives are uncles, aunts, nephews, nieces, grandparents, grandchildren, half siblings, and double cousins. Third degree relatives (12.5% shared DNA) include great-grandparents, great-grandchildren, grand-uncles, grand-aunts, first cousins, half-uncles, half-aunts, half-nieces, and half-nephews. ACMG indicates American College of Medical Genetics; Htx, heart transplantation; and LVAD, left ventricular assist device.

## METHODS

See the Supplemental Material for the complete methods. All couples were counseled by a clinical geneticist at the Maastricht Universitair Medische Centrum+ and enrolled in the diagnostic PGT procedure (licensed by the Dutch Ministry of Health, Welfare and Sport CZ-TSZ-291208) after giving informed consent that their data concerning PGT can be used for evaluation of treatment and scientific publications. The data that support the findings of this study are available from the corresponding author on reasonable request.

## RESULTS

### Patient Population Referred for PGT

**Figure 2.**
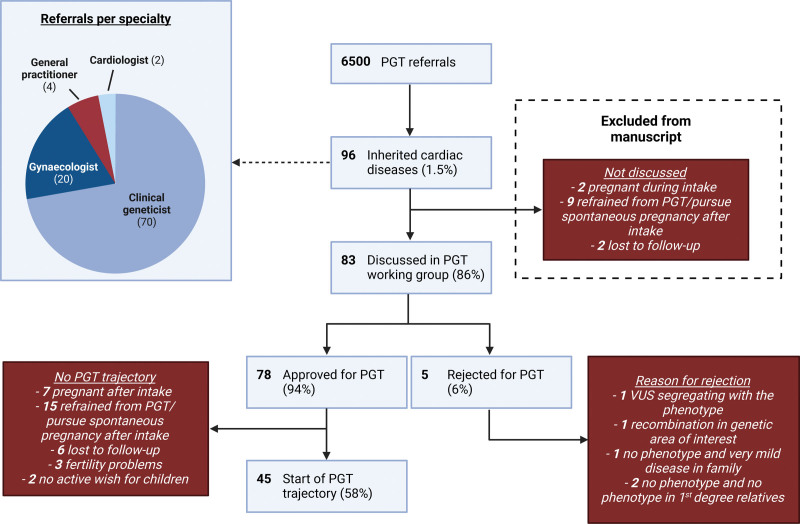
**Flow diagram of all couples that were referred for preimplantation genetic testing (PGT).** The indication of 13 couples was not discussed by the PGT multidisciplinary team because the couple decided not to pursue PGT after the intake counseling. Thirty-three indications were approved by the PGT multidisciplinary team, but eventually did not lead to the start of a PGT trajectory. VUS indicates variant of unknown significance.

**Figure 3.**
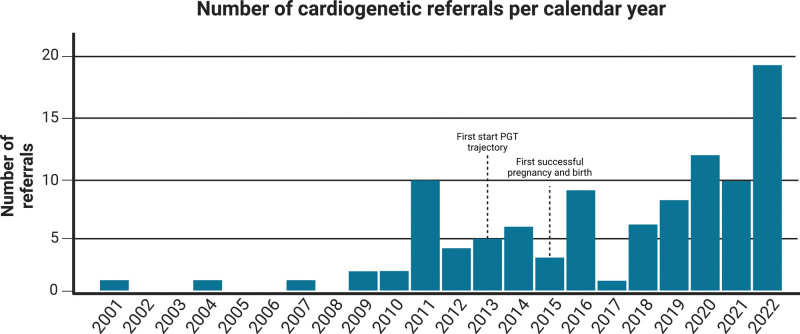
**History of preimplantation genetic testing (PGT) referrals for inherited cardiac diseases.** Although PGT is available since 1995, the first referral for PGT for inherited cardiac diseases was in 2001, but was only possible since 2009. Afterward, there was a steep increase in the number of referrals. ACM indicaes arrhythmogenic cardiomyopathy.

Since the first referral for PGT due to an inherited cardiac disease in 2001, a total of 96 couples were referred representing 1.5% of the total PGT referrals, of which 43% of the cardiogenetic referrals in the last 3 years (Figure 2). PGT for inherited cardiac disease was only possible since 2009, and since then the number of referrals has increased to ≈3.6% of the total PGT referrals (Figure 3). Most couples were referred to our center by a clinical geneticist. Thirteen of the 96 couples did not proceed with PGT after the intake counseling due to various reasons (Figure 2), and are therefore further excluded from the study (Table S1; Figure S1). In total, this cohort consisted of 83 couples with causal genetic variants in 19 different genes leading to 8 different cardiac phenotypes: dilated, hypertrophic, arrhythmogenic and noncompaction cardiomyopathy, long-QT syndrome, Brugada syndrome, catecholaminergic polymorphic ventricular tachycardia, and idiopathic ventricular tachycardia/fibrillation (Table 1; Tables S2 and S3).

**Table 1.**
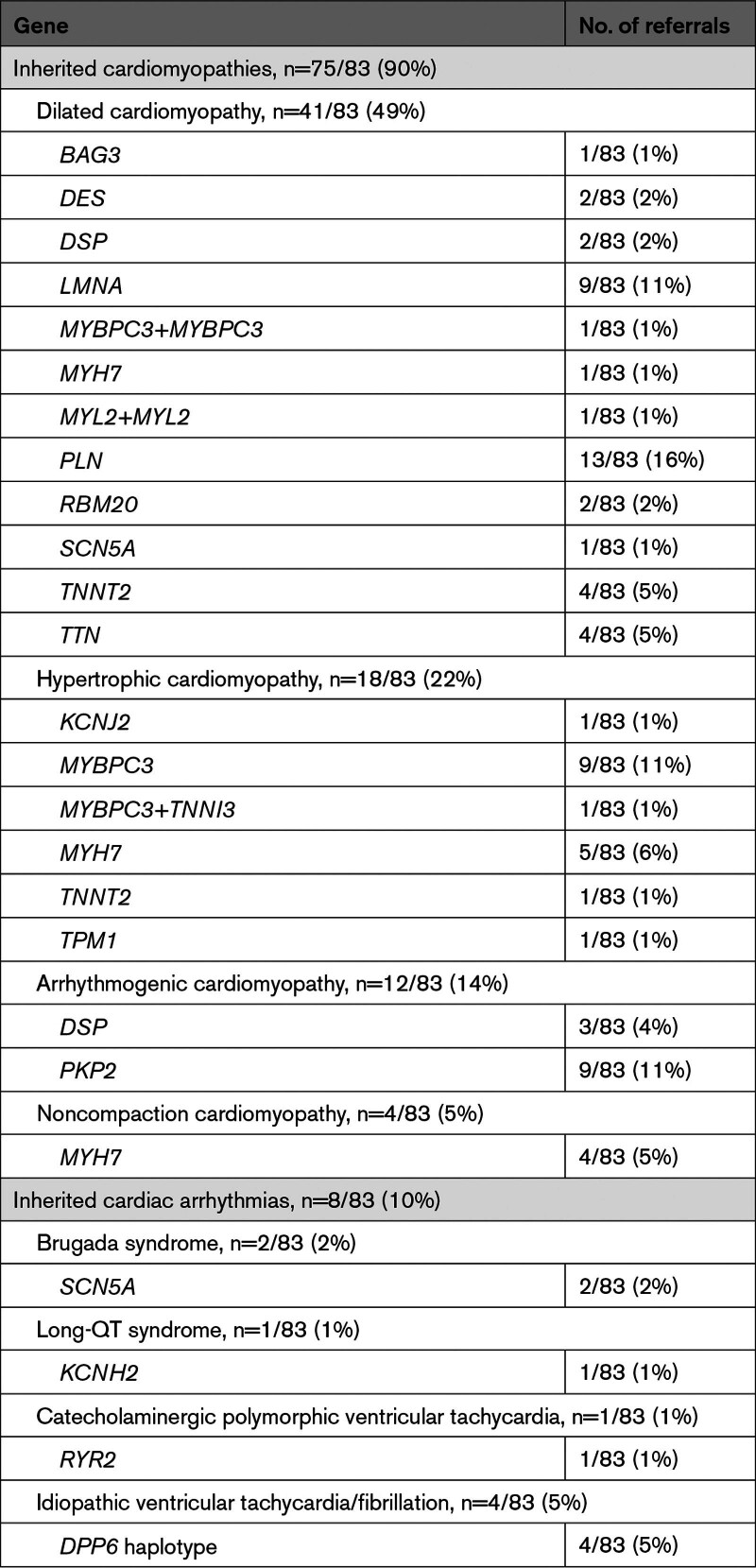
Phenotypes and Corresponding Genetic Landscape of Preimplantation Genetic Testing Referrals of Cardiac Diseases

Sixty index patients had a cardiac phenotype (70%) that manifested at a median age of 21 years (interquartile range, 17–39 years), and 27 were women. There was a high prevalence of heart transplantation at a young age in those with a phenotype (7%), and 38% of the affected gene carriers had ongoing life-threatening arrhythmias for which they received multiple appropriate shocks from their device.

Nine carriers did not have a family member with a phenotype. All others had a family history characterized by a young onset of disease, a high percentage of heart transplantation and high frequency of sudden cardiac death at a young age (Table 2).

**Table 2.**
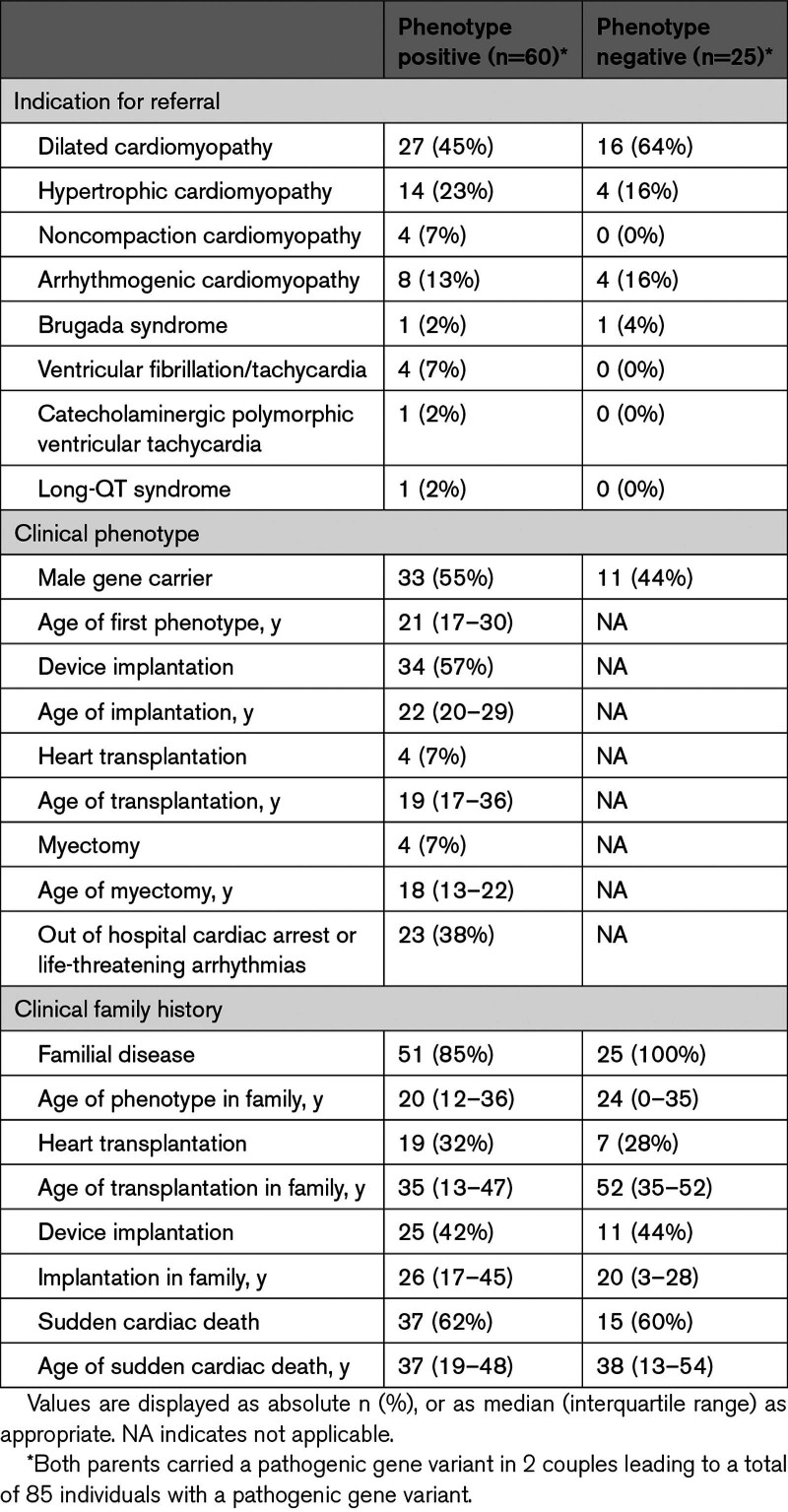
Baseline Characteristics of All Gene Carriers Who Were Referred for Preimplantation Genetic Testing (n=85)^*^

### Application of the Decision Model

**Figure 4.**
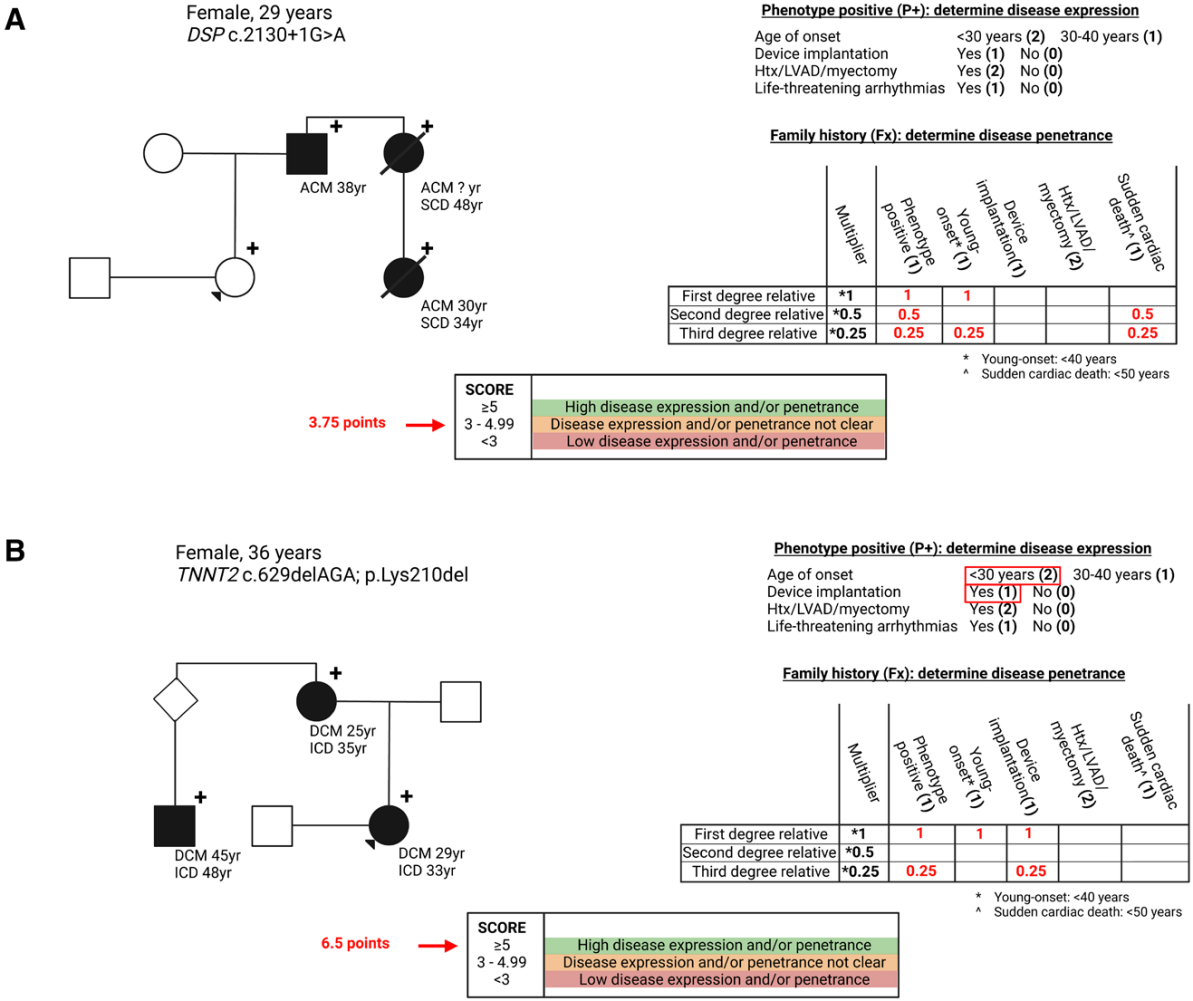
**Two examples of the application of step 2 of the model to included cases. A**, An asymptomatic 29-year-old woman who had the familial *DSP* variant. As she had no signs or symptoms herself, she scored 0 points on disease expression. Her father, aunt, and cousin were all affected and scored points on phenotype, young onset and sudden cardiac death (SCD). In total, 3.75 points were scored, concluding that the disease expression and penetrance is not clear. This is mainly due to the fact that the female carrier requisition PGT has no phenotype (yet). The advice is to discuss this specific case in a multidisciplinary meeting. **B**, A 36-year-old woman with a pathogenic *TNNT2* variant developed DCM at the age of 29 years for which she received an ICD implantation. Due to the young age of onset and device implantation, 3 points were scored for disease expression. An aunt and a maternal cousin both have the same variant and DCM for which they received an ICD. In total, 3.5 points were scored for disease penetrance, making a total of 6.5 points. The disease expression and penetrance of the *TNNT2* variant were high enough that the estimated risk reduction by PGT will be sufficient. Black symbols indicate affected individuals. The + symbol indicates the presence of the familial variant. The arrow indicates the individual requestion PGT. ACM indicates arrhythmogenic cardiomyopathy; DSP, desmoplakin; Htx, heart transplantation; ICD, implantable cardioverter defibrillator; and LVAD, left ventricular assist device.

**Figure 5.**
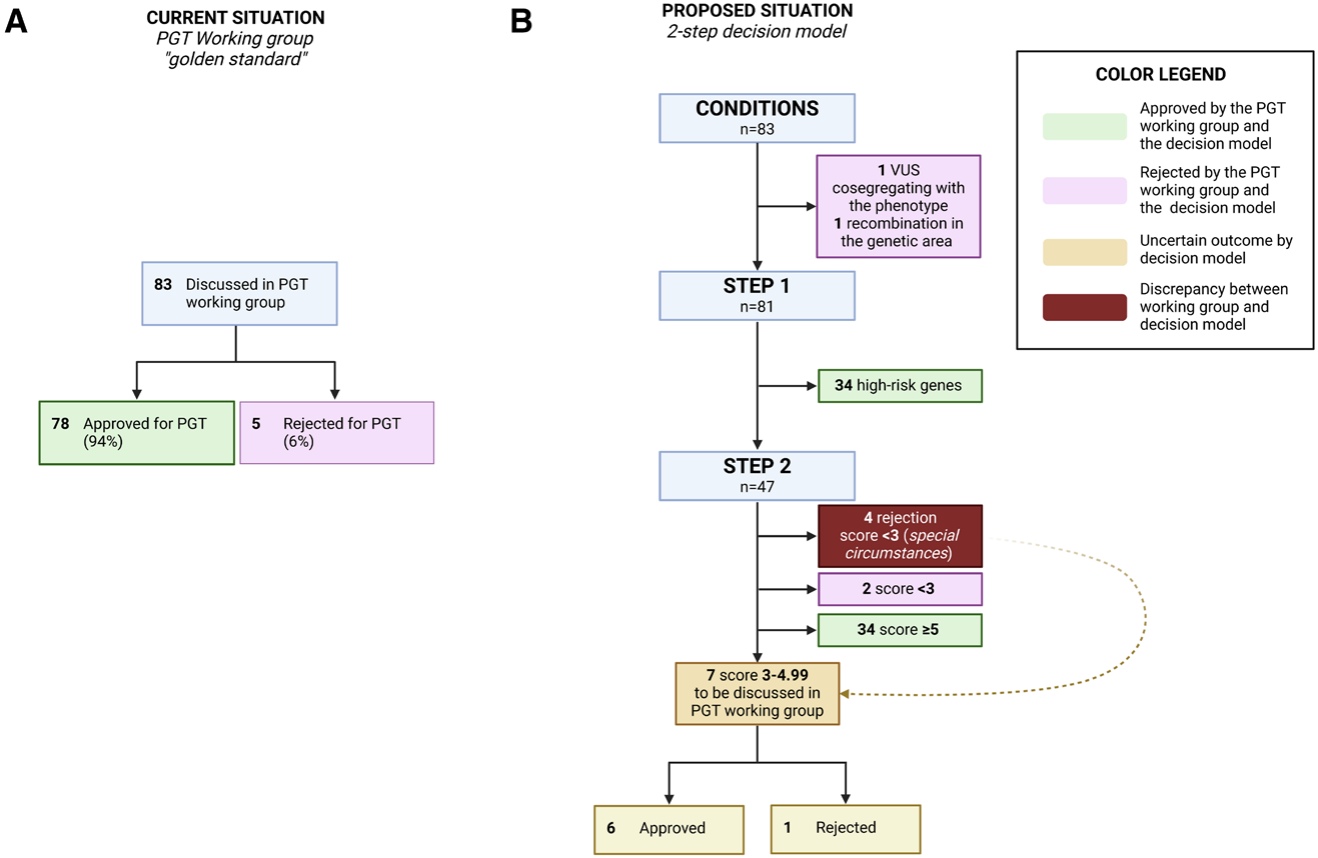
**Comparison between the decision of the preimplantation genetic testing (PGT) multidisciplinary team and our decision model.** In the current situation, all PGT indications for cardiac diseases are discussed by the PGT multidisciplinary team (**A**). Using the decision model, only 7 indications would need further discussion in the PGT multidisciplinary team (**B**). For the other 76 couples, the model was in accordance with the decision of the PGT multidisciplinary team in 72 cases (sensitivity 95%).

The decision model (Figure 1) was applied retrospectively to the 83 couples and is illustrated by 2 specific cases (Figure 4). The 4 predefined conditions were met for 81 couples (Figure 5B). For 1 couple who did not meet the conditions, familial segregation of variants in 2 genes was complex (condition 4). The male index with a mild dilated cardiomyopathy (DCM) and a pathogenic truncating *TTN* variant, also carried a variant of unknown significance (VUS) in *TNNC1*. His brother with both variants had severe DCM requiring a cardiac transplant at a young age. All other relatives who carried the VUS in *TNNC1* only had a mild DCM phenotype, and the carriers of the pathogenic *TTN* variant did not have a phenotype. It remains unknown what the influence of the VUS is in the disease expression and penetrance of the phenotype in this family. Thus, the condition for segregation of the variant with the phenotype was not met (Figure 1; condition 4). Furthermore, PGT for a VUS is not allowed. The second couple concerned a women who carried the risk *DPP6* haplotype for idiopathic ventricular fibrillation/ventricular tachycardia. However, genetic recombination took place in the area of interest in this family pedigree, thereby creating uncertainty what the contribution of the genetic region to the phenotype, and segregation within the family was not definite (Figure 1; condition 4). The remaining 81 couples could be scored in the 2-step model (Figure 5B).

#### Step 1 (Genotype)

Thirty-four couples (42%) had a pathogenic variant in one of the definitive high-risk genes: *PLN* (n=13), *LMNA* (n=9), *RBM20* (n=2), *BAG3* (n=1), and *PKP2* (n=9), and are thus eligible for PGT.

#### Step 2 (Phenotype)

The phenotype score was calculated for the remaining 47 couples. Of them, 34 had a score of 5 or higher (72%), 7 between 3 and 4.99 (15%), and 6 below 3 (13%) (Figure 5B).

### Comparison of the Model With the Outcome of the PGT Multidisciplinary Team

The outcome of the decision model was compared with the decision of the PGT multidisciplinary team (MT; Figure 5; Figure S2). The 2 couples who did not meet the conditions for PGT, as described in the above paragraph, were also rejected by the MT due to similar reasons.

In step 1, the approval of all carriers of a high-risk gene, as indicated by the model, was completely in line with the decision of the PGT MT. In step 2, 34/47 (72%) couples scored above 5 and would be eligible for PGT, in concordance with the decision of the PGT MT.

Six couples had a low score in the model and the conclusion based solely on the model would be ineligibility for PGT (Figure S2). Two couples with a low score (<3), were also rejected by the MT due to similar reasons. The other 4 rejected couples were approved by the PGT MT. Three of these 4 couples scored low in step 2 (0.99, 1, and 1.5), but were all primarily referred for a different pathogenic variant in a noncardiac gene for which PGT was a definitive indication: *BMPR2, AMER1*, and *BRCA1*. In all cases, the addition of the cardiogenetic variant as a secondary indication was approved by the PGT MT, although the cardiac indication would probably not have been approved as a single indication. The other discrepancy between the current model and the PGT MT involved a couple who was in an in vitro fertilization trajectory using donor semen from an anonymous donor and a complex history. In another couple using the same donor semen, a child with congenital DCM was born. Genetic testing in the affected child and the sperm donor revealed a pathogenic *MYH7* variant. Clinical information of the donor is lacking, and, therefore, the score remained at 2. Overall, 72 of the 76 decisions of the model were in accordance with the decision of the PGT MT (95%). Discrepancies between the model and the MT were all due to exceptional and complex results of genetic testing or family circumstances. Table S2 summarizes all monogenic indications and their corresponding decision that have been made by the PGT MT (Table S3). It should be noted that different couples with the same variant were given different decisions by the MT (eg, *DSP* p.[Arg941*]), showing the differences in penetrance among variants and the need for an objective model to score penetrance.

### Prospective Application of the Decision Model

To test the clinical utility of the decision model, we applied the model prospectively to all new referrals since July 2022, before discussion in the PGT MT. In total, there were 11 new referrals (Table 3). Following the decision model, 3 couples had a high score and would be approved, 1 had a low score and was rejected in the absence of other nonphenotypic factors that could alter the decision. Three couples had an intermediate score in step 2 and were discussed in detail in the PGT MT. Another couple had 2 pathogenic variants (*MYBPC3* and *PLN*). The *PLN* variant (c.40_42del) is a high-risk variant and was approved, but the addition of *MYBPC3* as a second indication was evaluated by the PGT MT. Two couples with a pathogenic *PKP2* variant had an intermediate score but were approved due to the high risk and penetrance associated with *PKP2.* One couple who were both carriers of a truncating *TTN* variant without a phenotype, had an earlier child that inherited both variants and died due to severe congenital heart disease. The model is not suitable for autosomal recessive disorders, but the indication was approved by the PGT MT. The prospective application of the decision model shows that utility in clinical practice is high, and provides quantifiable argumentation to assist decision-making.

**Table 3.**
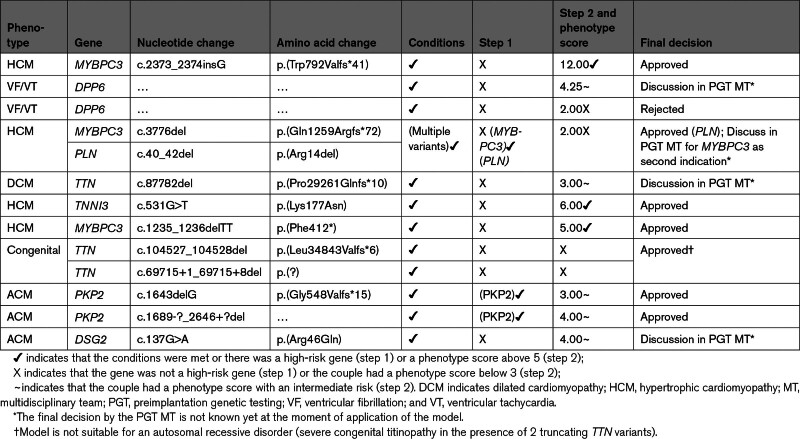
Application of the Decision Model on New Referrals Since July 2022

## DISCUSSION

We provide an overview of PGT for inherited cardiac diseases from an experienced national referral PGT center, where we observe a steep increase in the number of PGT referrals for inherited cardiac diseases. We developed a 2-step decision model to estimate the disease penetrance and expression of inherited cardiac disease in offspring, with a high sensitivity (95%) and strong utility in clinical decision-making, thereby estimating the eligibility for PGT. The decision model provides a transparent and uniform method to evaluate PGT referrals and select couples with a high risk of disease for their offspring. The model should always be combined with the personal context of an individual couple, thereby integrating the objective model with the personal view of a couple. It can be implemented in future guidelines regarding PGT inclusion for couples with inherited cardiac diseases, such as genetic cardiomyopathies and arrhythmias.

### Need for Guidance of PGT for Inherited Cardiac Diseases

Although the number of referrals in the past years was low, it is to be expected that the observed increase in the number of PGT referrals for inherited cardiac diseases will continue in the upcoming years, underscoring the need for specific guidelines and policy. Cardiogenetic disease is usually late onset with variable phenotypes and genetic contributions, complicating the decisions, which couples truly have a high risk of severely affected offspring. It is also becoming more evident that most cardiogenetic diseases are oligogenic or polygenic.^8^ As PGT aims for high-risk reduction in offspring, approval for PGT is less obvious for all requests, because a PGT trajectory in a family with low penetrance of an individual pathogenic variant will probably lead to a high-residual risk.^9^ We developed a robust and easily usable inclusion model that identifies which couples will have the highest risk reduction and will thus provide them and their doctors a quick and clear decision to proceed with PGT or not, while also optimizing the cost-benefit efficiency of this specialized procedure, and preventing unnecessary treatments.

### Clinical Implications of Genetic Testing in Cardiac Diseases

Currently, the finding of a pathogenic gene variant has mostly impact on the family of the patient as it will enable the possibility for genetic and cardiac screening in family members at risk.^4,5,10,11^ As such, genetic testing in family members can also identify younger (asymptomatic) relatives for whom PGT could be an option. The latest guidelines from the American College of Cardiology and AHA also highlight the impact of a genetic variant on the reproductive options of carriers, both patients and their family members.^4–7^ After the finding of a (likely) pathogenic variant, reproductive options, such as PGT, should be discussed to provide equal opportunities for all patients and their relatives. Awareness surrounding the reproductive possibilities by patients and medical specialists is the first step toward increased accessibility to PGT for all patients.

Remarkably, the clinical phenotype of the patients in our cohort was more severe compared with the average patient with an inherited cardiac disease.^12^ The mean age of disease onset was 21 years, compared with 52 years in an average genetic cardiomyopathy cohort. Also, the percentage of heart transplantations (7%), device implantations (57%), and life-threatening arrhythmias (38%) is extraordinary high in this age group. This finding suggests that there is a referral bias toward severely affected couples. Also, the self-selection of couples before referral may play a role, meaning that only couples with severe disease expression may actively ask for reproductive options such as PGT. The referral bias probably explains the high sensitivity of the model to predict the decision of the MT, because the less severely affected couples who are improved by the MT due to other considerations are underrepresented.

### Future Implementation and Policy Surrounding PGT for Inherited Cardiac Diseases

We acknowledge that the policy and accessibility to PGT varies among countries and continents. Almost all procedures in cardiology and clinical genetics are well protocolized, and guidelines on diagnosis and therapy are being updated according to the latest literature on a regular basis. In contrast, literature on PGT for inherited cardiac diseases is scarce.^3,13^ Our experience as a national PGT center, and the proposed decision model can be valuable in designing guidelines regarding PGT. Although we showed the clinical utility of the decision model in routine daily practice, it still needs to be validated in external, international cohorts. In addition, several outstanding questions should be addressed, for example, how to implement decision-making for multiple indications. We described multiple couples who requested PGT for >1 indication, complex results of genetic testing, or complex family situations. We conclude that in all these situations, the decision model should still be applied (ie, in the case of 2 autosomal dominant (cardiogenetic) indications for both indications separately), but we also recommend to discuss these complex cases in a multidisciplinary team.^14^ Also, the model can underestimate the risk in small families, where there are limited possibilities for segregation of a genetic variant. In such cases, discussion in a MT might also be considered.

The list of high-risk genes is likely to expand and be edited over time with increasing evidence and further insight into genotype-phenotype correlations and should be seen as a dynamic list on which the suggested 6 genes are a first proposal. The list can even be further specified including specific variants and type of variants (eg, truncating versus missense). Future possibilities to measure the polygenic risk score for cardiac diseases can provide additional information in estimating the potential risk reduction of PGT in families.^8^ Especially since an approved variant in a certain gene is no guarantee that the specific variant will also be approved in other families (or even within families; Table S3), because the penetrance can be vary between families. Future studies are necessary to integrate an individuals’ polygenic risk score into the model. In addition, as general knowledge on gene-specific penetrance is rapidly expanding, these public data can be integrated in the model to further fine tune the estimated penetrance within a family.

The described approach for gene variants with incomplete penetrance and PGT can also be translated to other genetic diseases in the future, using other disease-specific clinical variables.

### Limitations of the Study

The data presented in this article was gathered from a single national PGT center, representing the policy and PGT accessibility of a specific country. For example, in some countries, it can be argued to perform PGT for a suspicious VUS, although this is not the case in the Netherlands, and the model does not reflect this possibility. The rationale for risk reduction of inherited cardiac disease by PGT, however, is not bound to the area. The model can provide an objective measure of disease penetrance and expression in a family thereby guiding medical professionals. This is also relevant for those working in a country where PGT is largely self-funded, as it can provide an estimate of risk reduction. The model is based on available published guidelines and risk prediction models, and can therefore be incomplete. The model should be reevaluated with the advance of genotype-phenotype associations. Finally, the model should be prospectively evaluated in external cohorts before broad clinical implementation can be pursued.

### CONCLUSIONS

The number of PGT requests for inherited cardiac diseases is increasing fast underscoring the need for international guidelines. We propose a decision model that can help select those couples for PGT who will have the highest risk reduction for their progeny, which is now implemented in our national PGT center.

## ARTICLE INFORMATION

### Acknowledgments

All figures were created with biorender with the appropriate license.

### Sources of Funding

Dr Verdonschot is supported by a Dutch Heart Foundation Dekker, Clinical Scientist grant (03-005-2022-0040) and an Academic Fund by the Maastricht Universitair Medische Centrum+ (00499).

### Disclosures

None.

### Supplemental Material

Supplemental Methods

Tables S1–S3

Figures S1 and S2

References 15–28

## Supplementary Material


