# Supplementary material for: Clinical Guideline for Preimplantation Genetic Testing in Inherited Cardiac Diseases
Source: Circ Genom Precis Med. 2024 Mar 22;17(2):e004416. doi: 10.1161/CIRCGEN.123.004416 (PMC11019983; doi:10.1161/CIRCGEN.123.004416)
Supplement: Supplementary file 1 [file hcg-17-e004416-s001.pdf]

SUPPLEMENTAL MATERIAL

Supplemental Methods **Page 2.**

Table S1. **Page 10.**
Baseline characteristics of all gene carriers who were referred for PGT and did not proceed with the PGT trajectory after the first intake (n=13).

Table S2. **Page 11.**
Overview of all monogenic indications associated with inherited cardiac disease that are discussed in the PGT multidisciplinary team and their outcome.

Table S3. **Page 12.**
Overview of all (likely) pathogenic variants associated with inherited cardiac disease that are discussed in the PGT multidisciplinary team and their outcome.

Figure S1. **Page 14.**
Phenotypes and corresponding genetic landscape of PGT referrals of cardiac diseases that did not continue with the procedure after the first intake.

Figure S2. **Page 15.**
Application of step 3 to all couples and corresponding comparison to the eventual decision of the PGT multidisciplinary team.

SUPPLEMENTAL METHODS

Study population

All couples with an inherited cardiac disease, with the exception of congenital heart diseases, who were referred for PGT to the national PGT center in the Netherlands (Maastricht University Medical Center (Maastricht UMC+)) were included in this study. Couples at risk for congenital cardiac anomalies were excluded. All couples were counseled by a clinical geneticist at the Maastricht UMC+ and enrolled in the diagnostic PGT procedure (licensed by the Dutch Ministry of Health, Welfare and Sport CZ-TSZ-291208) after giving informed consent that their data concerning PGT can be used for evaluation of treatment and scientific publications. In the Netherlands, there is only one center that has the license to perform PGT, therefore all patients were seen in Maastricht. Clinical and genetic information was reviewed thoroughly from the electronic medical files. Detailed clinical information of the proband and the family history of cardiac related disease and sudden cardiac death was obtained by pedigree analysis during the intake counseling. If necessary, additional information of the prospective parent and family members was requested from their treating cardiologist.

Clinical procedure

After counseling at the department of clinical genetics at the Maastricht UMC+, couples could decide to proceed with PGT, or to decline from further procedure. PGT is covered by the health insurance of all Dutch inhabitants, meaning that the couple does not have to pay for the procedure. Currently, if a couple with an inherited cardiac disease decides to proceed, their indication is obliged to be discussed in a local PGT multidisciplinary team (MT) constituting of clinical geneticists, genetic counselors, case managers, gynecologists, ethicists, embryologists, and molecular specialists. If necessary, other specialists, such as a cardiologist or psychologists can be consulted. Every individual couple is evaluated in great detail by

multiple members of the PGT MT. The indication is either approved or rejected by the PGT MT, based on the clinical and genetic data of the patient and his or her family members.

In the current study, we developed a decision model to objectify disease penetrance of genetic variants and accelerate decision making for couples requesting PGT for inherited cardiac diseases. The outcomes of the model were compared in retrospect, to the decisions made by the PGT MT. The decision of the PGT MT was used as the 'gold standard'. Application of the developed decision model was performed in a blinded fashion by two independent researchers: the outcomes of the PGT MT were not known to the researchers at the moment of application of the model, with a complete accordance between the two researchers.

Development of the decision model

We were not able to perform a statistical approach to develop a decision model based on the gold standard due to two reasons: 1. the decision of the MT (gold standard) was reached based on phenotype and penetrance, but also psychological impact and technical implications were taken into account. Our model only focuses on phenotype. 2. we have a very biased study population, as most referrals are severely affected, young individuals. Therefore this population is not suited to estimate disease penetrance in general.

In the decision model we incorporated the clinical expression (*e.g.* age at onset, disease phenotype, severity), and penetrance of a pathogenic variant (genotype) of carriers and their family members to estimate the risk of overt cardiac disease for offspring and thus eligibility for PGT (**Figure 1**). Specifically, the components, conditions and questions in the decision model were developed by incorporating both current variant classification as well as current clinical cardiac disease guidelines (gene curation [15-18], variant classification [19,20], and diagnosis, management and prevention of cardiomyopathy and sudden cardiac death [4,5,11]). In addition,

clinical parameters from cardiac gene-specific risk prediction models were incorporated [21-23]. The decision model is therefore based on a combination of clinical risk prediction models, and published evidence of genetic variance in inherited cardiac diseases.

Firstly, we defined the conditions that must be met before the model can be applied. In general, it is crucial that the genetic diagnosis in the index is solid and comprehensive cardiac gene panel diagnostics has been performed. Therefore, we defined four conditions.

1. Complete genetic testing of the index patient using a panel composed of at least all robust disease-associated genes is mandatory to exclude a second pathogenic gene variant. A single gene analysis will not suffice in affected individuals for PGT inclusion. If clinically unaffected family members carrying a familial pathogenic variant opt for PGT, then the closest affected patient in the family must have had complete genetic testing.
2. The gene for which PGT is requested must be curated as 'Strong' or 'Definitive' associated with the observed phenotype. Gene-disease validity assessment is performed by gene curation expert panels of the clinical genome resource (ClinGen) consortium for most cardiac phenotypes [15-18]. Evidence-based assessment of genes related to cardiac phenotypes are published by the corresponding expert panels, or can be found in the online database (www.clinicalgenome.org).
3. The detected variant must be classified as 'Likely pathogenic' (class 4) or 'Pathogenic' (class 5) using the ACMG/AMP criteria [19]. A 'Variant of unknown significance' is not eligible for PGT inclusion.
4. Genetic testing of as many as possible affected and first-degree family members is recommended to have sufficient information on variant segregation with the phenotype in the family, or a *de novo* occurrence. In addition, genetic testing of

first-degree family members is recommended for the technical development of PGT diagnostic test.

After these conditions are met, the two steps of the decision model can be applied (**Figure 1**). *Step 1: selection based on genotype* – We performed a literature search on all genes that are 'definitive' associated with a cardiac disease according to the ClinGen consortium to collect evidence on penetrance and/or prognosis of variants in these genes. A specific number of genes is almost always associated with a highly penetrant and/or severe form of cardiac disease, without clinical heterogeneity: *PLN* (c.40_42del founder variant), *LMNA, BAG3, RBM20,* and truncating variants in *FLNC* for dilated cardiomyopathy (DCM), and *PKP2* for arrhythmogenic cardiomyopathy (ACM) (**Figure 1,** *step 1*) [4,21-28]. A (likely) pathogenic variant in one of these six genes should be considered for PGT inclusion in any carrier, irrespective of the familial phenotype. The high-risk genes currently included in step 1 are selected based on 1) availability of multicenter studies revealing the adverse outcome of these genotypes, and 2) the inclusion of these genes in guidelines for the treatment of heart failure, ventricular arrhythmias, and prevention of sudden cardiac death. All genes were reviewed by a multi-disciplinary team, leading to the adjudication of these six genes as high-risk.

Step 2: review of the phenotype – (Likely) pathogenic variants in other genes such as *MYH7, MYBPC3* and *SCN5A* can lead to a severe cardiac phenotype, but the clinical presentation and penetrance vary strongly among families. For those genes, the model can be applied to estimate disease penetrance and expression in the family using the clinical data of the carrier(s) and their family. Scored items and the corresponding points that can be scored are: age of onset (<30 years (2); 30-40 years (1)), device implantation (1), heart transplantation (2), left ventricular assist device (2), septal myectomy (2), life-threatening arrhythmias (1), and sudden cardiac death (1). If the referred gene carrier has a phenotype, the items are scored for him or her

(**Figure 1,** *step 2*). These clinical parameters are also scored for their affected relatives, with the addition of sudden cardiac death under the age of 50 years. The same clinical parameter can be scored multiple times if multiple family members are affected. Due to the contribution of a possible polygenic background necessary for disease penetrance of the primary familial variant, a multiplier is used for affected second- (*0.5) and third-degree relatives (*0.25), to correct for dilution of this polygenic background in relatives further away from the gene carrier requesting PGT.

We propose the following thresholds for the final score of the model: if the final score of step two is 5 or higher, the disease penetrance and expression of the genetic variant in the family and thus the risk of severe expression in a future child, are considered high enough to justify PGT. When the score is between 3 and 4.99 (intermediate), the request should be discussed in a multidisciplinary team (MT). Below a score of 3 (low), disease penetrance is low and PGT should in general not be considered, in the absence of other more subjective arguments.

Statistical calculation of proposed thresholds

The average scores of all included 83 couples was calculated (mean 5.96±2.53; median 5.5 with IQR 4 – 7.5), and divided based on approval or rejection by the PGT multidisciplinary team (the gold standard):

a. Approved: 6.03±2.47; median 5.75 with IQR 4.75 – 7.5
b. Rejected: 4.90±3.55; median 4.00 with IQR 1.63 – 8.63

The phenotypes of all 83 couples were evaluated in relation to the score of the model and the decision by the PGT MT. Two couples that were rejected because of technical difficulties of the procedure, had a score of 8.25 and 9.00, respectively. Indicating that these indications

normally would have been approved. In addition, there were also couples that had low scores, but were approved due to exceptional circumstances (*eg.* PGT for more than one gene, the presence of high-risk genes in the absence of a clear phenotype). In the absence of these circumstances, these indications would not have been approved by the MT. We excluded these couples that would have reached a different decision solely based on the phenotype and genetic information. Afterwards, we re-calculated the scores:

a. Approved: 7.05±2.37; median 6.75 with IQR 5 – 8.75

b. Rejected: 2.42±1.38; median 1.75 with IQR 1.5 – 3

These scores were used to define the thresholds: the lower quartile of the approved was the threshold for 'high penetrance' (score of 5), the upper quartile of the rejected was the threshold for 'low penetrance' (score of 3), and the group in between (score between 3 and 5) was defined as the intermediate group.

<u>Statistical analysis</u>

Variables are displayed as numbers (percentage), mean ± standard deviation or median with interquartile range (IQR) as appropriate. Statistical analysis was performed using SPSS 23.0 (IBM Corp., Armonk, NY, USA) software.

Table S1. Baseline characteristics of all gene carriers who were referred for PGT and did not proceed with the PGT trajectory after the first intake (n=13). These patients were not included in the main paper.

	Phenotype positive (n=9)	Phenotype negative (n=4)
Indication for referral		
Dilated cardiomyopathy	1 (11%)	0 (0%)
Hypertrophic cardiomyopathy	3 (33%)	2 (50%)
Non-compaction cardiomyopathy	0 (0%)	2 (50%)
Arrhythmogenic cardiomyopathy	0 (0%)	0 (0%)
Brugada syndrome	2 (22%)	0 (0%)
Ventricular fibrillation/tachycardia	0 (0%)	0 (0%)
Catecholaminergic polymorphic ventricular tachycardia	0 (0%)	0 (0%)
Long QT syndrome	3 (33%)	0 (0%)
Clinical phenotype		
Male gene carrier	5 (56%)	3 (75%)
Age of phenotype, years	22 [15 - 27]	NA
Device implantation	3 (33%)	NA
Age of implantation, years	22 [18 - 22]	NA
Heart transplantation	1 (11%)	NA
Age of transplantation, years	40	NA
Myectomy	1 (11%)	NA
Age of myectomy, years	17	NA
Out of hospital cardiac arrest or life-threatening arrhythmias	2 (22%)	NA
Clinical family history		
Familial disease	5 (56%)	4 (100%)
Age of phenotype in family, years	40 [15 – 47]	33 [8 - 38]
Heart transplantation	0 (0%)	2 (50%)
Age of transplantation in family, years	NA	20 [4 – 20]
Device implantation	3 (33%)	2 (50%)
Implantation in family, years	35 [16 – 35]	30
Sudden cardiac death	2 (22%)	2 (2%)
Age of sudden cardiac death, years	48 [35 – 57]	48 [9 – 60]

Values are displayed as absolute number (percentage), or as median [interquartile range] as appropriate.
NA indicates not applicable.

Table S2. Overview of all monogenic indications associated with inherited cardiac disease that are discussed in the PGT multidisciplinary team and their outcome. Couples with multiple variants are not included (n=3).

Gene	Phenotype	Approved by PGT multidisciplinary team	PGT trajectory started	Number of couples (approved/rejected/started)
BAG3	DCM	Yes	Yes	1 / 0 / 1
DES	DCM	Yes	Yes	2 / 0 / 1
DPP6 haplotype	VF/VT	Yes	No	3 / 1 / 0
DSP	DCM	Yes	Yes	4 / 1 / 3
KCNH2	LQT	Yes	Yes	1 / 0 / 1
KCNJ2	HCM	Yes	Yes	1 / 0 / 1
LMNA	DCM	Yes	Yes	9 / 0 / 4
MYBPC3	HCM	Yes	Yes	7 / 2 / 5
MYH7	DCM	Yes	Yes	1 / 0 / 1
MYH7	HCM	Yes	Yes	5 / 0 / 1
MYH7	NCCM	Yes	Yes	4 / 0 / 3
PKP2	ACM	Yes	Yes	9 / 0 / 5
PLN	DCM	Yes	Yes	13 / 0 / 8
RBM20	DCM	Yes	Yes	2 / 0 / 2
RYR2	CPVT	Yes	Yes	1 / 0 / 1
SCN5A	DCM	Yes	No	1 / 0 / 0
SCN5A	Brugada	Yes	No	2 / 0 / 0
TNNT2	DCM	Yes	Yes	4 / 0 / 2
TNNT2	HCM	Yes	No	1 / 0 / 0
TPM1	HCM	Yes	No	1 / 0 / 0
TTN	DCM	Yes	Yes	3 / 1 / 3

* Overall, 5 indications were rejected by the PGT multidisciplinary team (DPP6 haplotype, DSP, MYBPC3, TTN). The specific variants and corresponding classification can be found in **Supplemental Table 3**.
Abbreviations: HCM indicates hypertrophic cardiomyopathy; VF/VT, ventricular fibrillation/ventricular tachycardia; DCM, dilated cardiomyopathy, LQT, long QT syndrome; NCCM, non-compaction cardiomyopathy; ACM, arrhythmogenic cardiomyopathy; CPVT, catecholaminergic polymorphic ventricular tachycardia.

Table S3. Overview of all (likely) pathogenic variants associated with inherited cardiac disease that are discussed in the PGT multidisciplinary team and their outcome.

Gene	Phenotype	Nucleotide change	Amino acid change	ACMG classification	Decision PGT multidisciplinary team
BAG3	DCM	c.915dup	p.(Met306Hisfs*13)	LP	Approval
DES	DCM	c.1024A>G	p.(Asn342Asp)	P	Approval
DES	DCM	c.735+1G>A	p.(?)	P	Approval
DPP6 haplotype	VF/VT	-	-	P	Approval (x3); Rejected (x1)
DSP	ACM	c.2130+1G>A	p.(?)	LP	Approval (x3)
DSP	DCM	c.2821C>T	p.(Arg941*)	P	Approval (x1); Rejected (x1)
KCNH2	LQT	c.1838C>T	p.(Thr613Met)	P	Approval
KCNJ2	HCM	c.652C>T	p.(Arg218Trp)	P	Approval
LMNA	DCM	c.1130G>T	p.(Arg377Leu)	P	Approval (x4)
LMNA	DCM	c.1130G>A	p.(Arg377His)	P	Approval
LMNA	DCM	c.992G>A	p.(Arg331Gln)	P	Approval
LMNA	DCM	c.568C>T	p.(Arg190Trp)	P	Approval
LMNA	DCM	c.1493G>A	p.(Trp498*)	P	Approval
LMNA	DCM	c.(?_274)_(352_?)del	p.(?)	LP	Approval
MYBPC3 + *MYBPC3*	DCM	c.2827C>T + c.2373_2374insG	p.(Arg943*) + p.(Trp792Valfs*41)	P + P	Approval
MYBPC3	HCM	c.2373_2374insG	p.(Trp792Valfs*41)	P	Approval (x3); Rejected (x1)
MYBPC3	HCM	c.1831G>A	p.(Glu611Lys)	P	Approval
MYBPC3	HCM	c.3776delA	p.(Gln1259fs)	P	Approval
MYBPC3	HCM	c.2827C>T	p.(Arg943*)	P	Approval
MYBPC3	HCM	c.1235_1236delTT	p.(Phe412*)	P	Approval
MYBPC3	HCM	c.2993A>G	p.(Gln998Arg)	LP	Approval
MYBPC3 + *TNNI3*	HCM	c.481C>T + c.297_311del15insCG	p.(Pro161Ser) + p.(Gln99Hisfs*12)	LP + P	Approval
MYH7	DCM	c.5754C>G	p.(Asn1918Lys)	P	Approval
MYH7	HCM	c.1816G>A	p.(Val606Met)	P	Approval
MYH7	HCM	c.2156G>A	p.(Arg719Gln)	P	Approval (x2)
MYH7	HCM	c.1357C>T	p.(Arg453Cys)	P	Approval
MYH7	HCM	c.1063G>A	p.(Ala355Thr)	P	Approval
MYH7	NCCM	c.732+1G>A	p.(?)	P	Approval
MYH7	NCCM	c.4169+2T>A	p.(?)	LP	Approval
MYH7	NCCM	c.495G>A	p.(Met165Ile)	P	Approval (x2)
MYL2 + *MYL2*	DCM	c.376C>T + c.376C>T	p.(Gln126*) + p.(Gln126*)	P + P (AR)	Approval
PKP2	ACM	c.1211dupT	p.(Val406Serfs*4)	P	Approval
PKP2	ACM	c.2386T>C	p.(Cys796Arg)	P	Approval (x4)
PKP2	ACM	c.917_918delCC	p.(Pro306fs)	P	Approval
PKP2	ACM	c.397C>T	p.(Gln133*)	P	Approval (x2)
PKP2	ACM	c.1211dupT	p.(Val406Serfs*4)	P	Approval
PLN	DCM	c.40_42del	p.(Arg14del)	P	Approval (x13)

RBM20	DCM	c.1900C>T	p.(Arg634Trp)	P	Approval
RBM20	DCM	c.1910G>A	p.(Ser637Asn)	LP	Approval
RYR2	CPVT	c.1258C>T	p.(Arg420Trp)	P	Approval
SCN5A	Brugada	c.5228G>A	p.(Gly1743Glu)	P	Approval
SCN5A	Brugada	c.5243G>T	p.(Gly1748Asp)	LP	Approval
SCN5A	DCM	c.2482C>T	p.(Leu828Phe)	LP	Approval
TNNT2	DCM	c.392G>C	p.(Arg131Pro)	LP	Approval
TNNT2	DCM	c.629delAGA	p.(Lys210del)	P	Approval
TNNT2	DCM	c.421C>T	p.(Arg141Trp)	P	Approval (x2)
TNNT2	HCM	c.274C>T	p.(Arg92Trp)	P	Approval
TPM1	HCM	c.184G>C	p.(Glu62Gln)	P	Approval
TTN	DCM	c.42379C>T	p.(Arg14127*)	LP	Approval
TTN	DCM	c.48015_48016del	p.(Asp16007Profs*19)	LP	Approval
TTN	DCM	c.69325G>T	p.(Glu23109*)	LP	Rejected
TTN	DCM	c.80530delA	p.(Ser26844Valfs*29)	LP	Approval
LP indicates likely pathogenic; P is pathogenic, AR is autosomal recessive)					

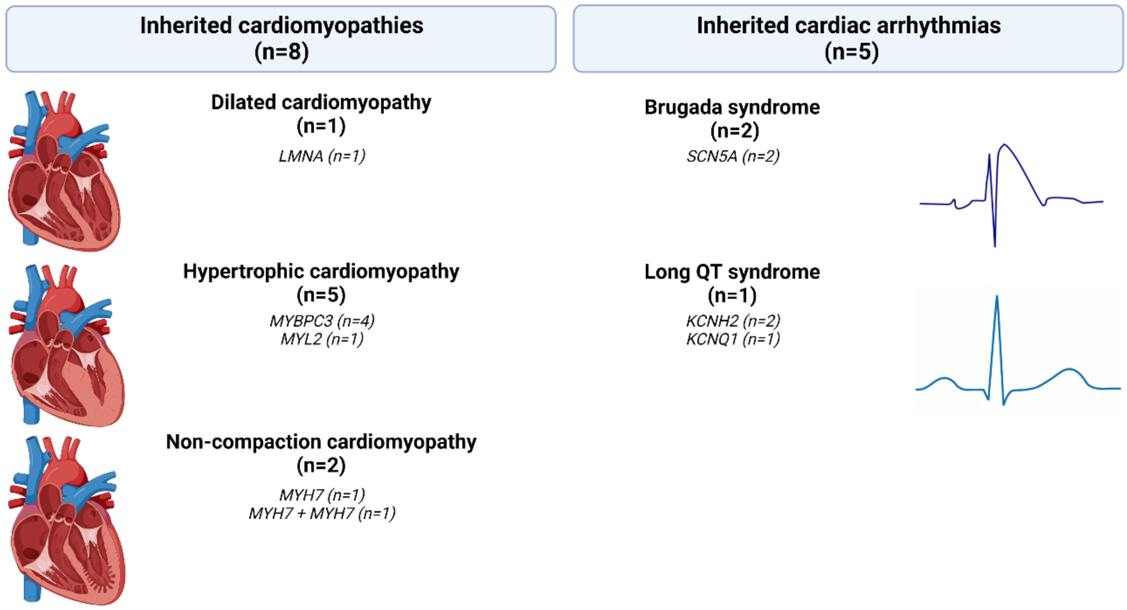

Figure S1. Phenotypes and corresponding genetic landscape of PGT referrals of cardiac diseases that did not continue with the procedure after the first intake. These couples and indications were not included in the manuscript as they were not discussed in the PGT multidisciplinary team.

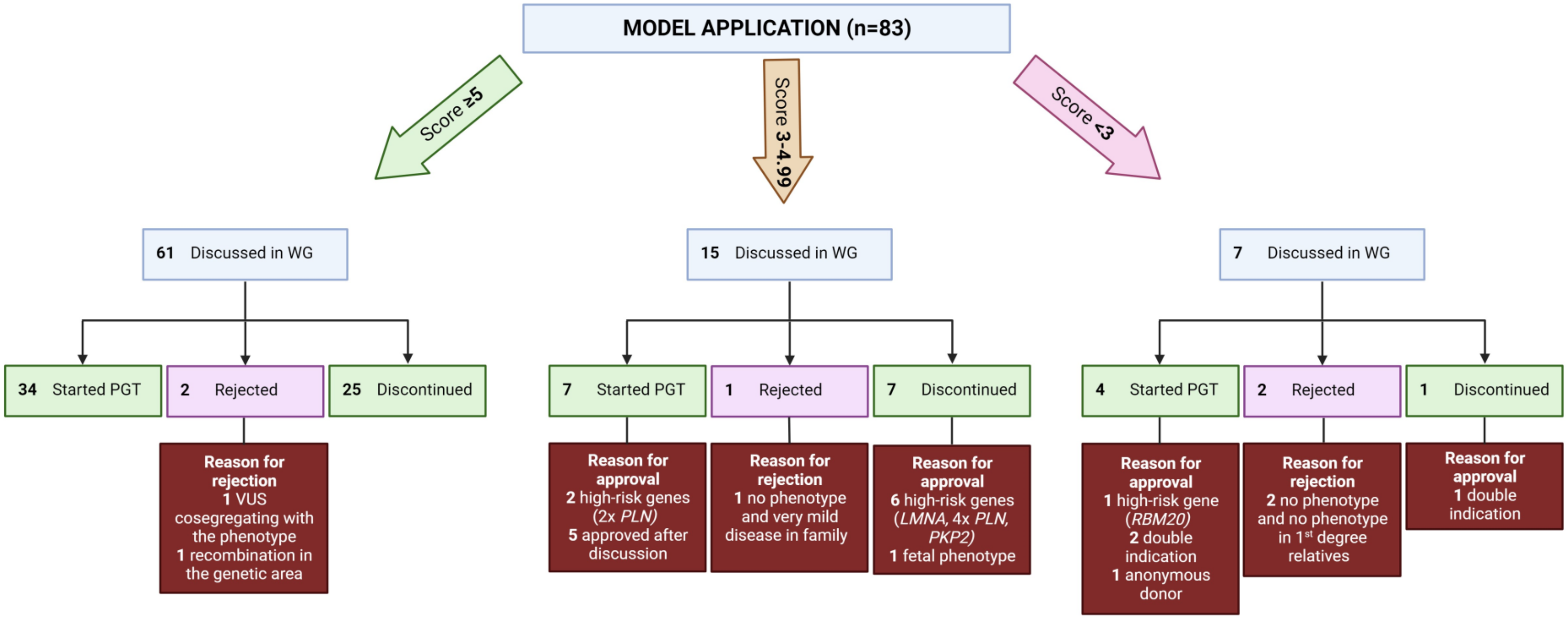

Figure S2. Application of step 3 to all couples and corresponding comparison to the eventual decision of the PGT multidisciplinary team. The green boxes show PGT indications that were approved by the PGT multidisciplinary team, the pink boxes indicate rejections by the multidisciplinary team. The dark red boxes below show the considerations for PGT approval in the intermediate and low risk group, or the reasons for rejection.
